# Intensive care unit to unit capacity transfers are associated with increased mortality: an observational cohort study on patient transfers in the Swedish Intensive Care Register

**DOI:** 10.1186/s13613-022-01003-x

**Published:** 2022-04-04

**Authors:** Fredric Parenmark, Sten M. Walther

**Affiliations:** 1 Centre for Research and Development, Uppsala University, Region Gävleborg, Gävle, Sweden; 2 Department of Anaesthesia and Intensive Care, Gävle Hospital, Gävle, Sweden; 3 Department of Medical and Health Sciences, Faculty of Health Sciences, Linköping University, Linköping, Sweden; 4 Department of Cardiothoracic Anaesthesia and Intensive Care, Linköping University Hospital, Linköping, Sweden

## Abstract

**Background:**

Transfers from one intensive care unit (ICU) to another ICU are associated with increased length of intensive care and hospital stay. Inter-hospital ICU transfers are carried out for three main reasons: clinical transfers, capacity transfers and repatriations. The aim of the study was to show that different ICU transfers differ in risk-adjusted mortality rate with repatriations having the least risk.

**Results:**

Observational cohort study of adult patients transferred between Swedish ICUs during 3 years (2016–2018) with follow-up ending September 2019. Primary and secondary end-points were survival to 30 days and 180 days after discharge from the first ICU. Data from 75 ICUs in the Swedish Intensive Care Register, a nationwide intensive care register, were used for analysis (89% of all Swedish ICUs), covering local community hospitals, district general hospitals and tertiary care hospitals. We included adult patients (16 years or older) admitted to ICU and subsequently discharged by transfer to another ICU. Only the first admission was used. Exposure was discharge to any other ICU (ICU-to-ICU transfer), whether in the same or in another hospital. Transfers were grouped into three predefined categories: clinical transfer, capacity transfer, and repatriation. We identified 15,588 transfers among 112,860 admissions (14.8%) and analysed 11,176 after excluding 4112 repeat transfer of the same individual and 300 with missing risk adjustment. The majority were clinical transfers (62.7%), followed by repatriations (21.5%) and capacity transfers (15.8%). Unadjusted 30-day mortality was 25.0% among capacity transfers compared to 14.5% and 16.2% for clinical transfers and repatriations, respectively. Adjusted odds ratio (OR) for 30-day mortality were 1.25 (95% CI 1.06–1.49 *p* = 0.01) for capacity transfers and 1.17 (95% CI 1.02–1.36 *p* = 0.03) for clinical transfers using repatriation as reference. The differences remained 180 days post-discharge.

**Conclusions:**

There was a large proportion of ICU-to-ICU transfers and an increased odds of dying for those transferred due to other reasons than repatriation.

**Supplementary Information:**

The online version contains supplementary material available at 10.1186/s13613-022-01003-x.

## Background

Intensive care beds are expensive and limited. The need for beds varies over time, and sometimes all beds are occupied, particularly on intensive care units (ICUs) where a high proportion of admissions are acute. When a critically ill patient needs admitting to a full ICU, the usual procedure is to either delay admission or create a temporary bedspace for a short period, while trying to free an intensive care bed by discharging another patient urgently to a general ward or to another ICU. This strategy comes at a cost since, firstly, premature discharge to a general ward may be associated with increased mortality [1] and, secondly, transfer to another ICU (ICU-to-ICU transfer) appears to be associated with increased total duration of intensive care and hospital stay, although unclear whether this is associated with increased mortality [2, 3].

Studies on ICU-to-ICU transfer are problematic since transfers are carried out for three main reasons that must be considered separately in the analyses. First, patients are transferred when there is need for specialised care that is not available in the admitting hospital (clinical transfer). Second, ICU patients are transferred to their home ICU after having undergone initial treatment at another unit (repatriation). Third, patients are transferred to make room for patients with more urgent need for intensive care when all ICU beds are occupied (capacity transfer). Furthermore, follow-up of transferred patients should preferably be carried out after discharge from ICU or hospital to capture important long-term effects on survival.

The present study is based on data from a large nationwide intensive care register, the Swedish Intensive Care Register (SIR), which registers the three principal reasons for transfer as well as long-term follow-up data. Our aim was to compare mortality between the three classes of ICU-to-ICU transfers with the assumption that mortality was lowest among repatriations.

## Methods

This was an observational cohort study on patients admitted to Swedish intensive care units (ICUs) from Jan 1st, 2016 to Dec 31st, 2018. Follow-up ended Sept 30th, 2019.

### Setting and participants

We used the Swedish Intensive Care Register (SIR) to identify eligible patients (see below). SIR is a national quality register which collects data from intensive care admissions in Sweden. Admissions to a few non-affiliated and paediatric ICUs were not included, leaving data from 75 ICUs for analysis (89% of all Swedish ICUs). The ICUs were located in local community hospitals (25 ICUs), district general hospitals (24 ICUs) and tertiary care hospitals (26 ICUs).

We included patients (16 years or older) admitted to ICU and subsequently discharged by transfer to another ICU. For patients with multiple admissions during the study period, we included the first admission only using the Swedish personal identity number for identification [4]. We excluded patients missing to follow-up (i.e. non-Swedes and a few individuals with concealed identity number, *n* = 714) or missing SAPS3 risk-adjustment data (*n* = 300 in 4 ICUs).

### Variables and definitions

The primary end-point, survival 30 days after discharge from the first ICU, and the secondary end-point, survival 180 days after discharge from the first ICU, were both determined by linking SIR to the Swedish Population Register.

Exposure was discharge to any other ICU (ICU-to-ICU transfer), whether in the same or in another hospital. Transfers are grouped by participating ICUs in three categories according to SIR guidelines: clinical transfer, capacity transfer, and repatriation. Clinical transfer is when the patient is transferred for specialised treatment or investigations not provided in the referring ICU. Capacity transfer is when a patient is transferred to make room for another patient with more urgent need for intensive care when all ICU beds are occupied. Repatriation is when a patient is transferred from the referring ICU to another ICU closer to the patient’s home.

Patient age, gender and admission and discharge times were retrieved from SIR which was the principal data source. The duration of ICU stay was calculated, as well as identification of discharges at night and during weekends. Night-time was defined as 10.00 PM to 6.59 AM and weekend as Saturday 0.00 AM to Sunday 23.59 PM [5]. We used the Simplified Acute Physiology Score (SAPS) 3 model to score chronic comorbidities and circumstances prior to admission, and reasons for admission and physiologic derangements on admission to ICU [6]. The score was subdivided into the original three boxes where Box 1 included comorbidities and time in hospital before ICU (age was deducted from Box1), Box 2 included circumstances on admission, and Box 3 included reasons for admission and physiological derangements on admission. Organ failure at discharge was calculated according to the Sequential Organ Failure Assessment (SOFA) score [7]. The score was based on clinical examination before discharge and blood samples obtained on the day of discharge. Missing individual organ scores were presumed normal (0 points). One primary and multiple secondary disease diagnoses were recorded by the attending physician at discharge from ICU according to SIR guidelines. In our analyses we used the primary diagnosis only. The International Classification of Diseases version 10 (ICD-10) was used to group patients into six principal disease groups (see Additional file 1: Table S1).

Data were recorded in raw format by each ICU and after local validation transferred to SIR for central validation (required data were present, entries were within prespecified limits, and inconsistencies and illogical entries were identified). If necessary, data were returned for correction and revalidation before being accepted and entered into the master database. In addition to a required comprehensive data set, SIR has a number of optional data sets, including SAPS3 and SOFA, which were used in the present study. While the SAPS3 set was used in all but a few ICUs (300 admissions in 4 ICUs had missing SAPS3 data), SOFA was used in 22 ICUs only. We used admissions from 75 ICUs with SAPS3 data and 22 ICUs with SAPS3 data and SOFA scores at discharge in our risk-adjusted analyses.

### Calculations and statistical methods

Descriptive data are presented as mean (95% confidence intervals, CI) or median (interquartile range, IQR) values and proportions (95% CI) as appropriate. Differences in crude survival were examined using the Kaplan–Meier estimate and the log-rank test.

The association between category of transfer and survival was analysed using univariable and two-level multivariable logistic regression models. The two-level approach was used to model intra-cluster correlation with ICUs treated as a random factor. The primary multivariable model was adjusted for age, gender, comorbidity, reasons for admission, circumstances and physiological derangements on admission as recorded in the SAPS3 model, duration of ICU stay and whether ICU discharge was during the night or weekend. All variables determined by expert opinion, prior experience and common use in critical care literature as relevant predictors of mortality. Additional candidate variables, not used, were completely decomposed SAPS3 score (instead of the partially broken-down score in this study), hospital category, admission time, day of the week and season of the year. All variables in our analyses were related to admission and treatment in the referring ICU, no variables were collected during transport or in the receiving ICU.

In a secondary model, in addition to the other variables, the SOFA score was used at discharge using observations from the 22 ICUs using SOFA scores. We also performed three sensitivity analyses in different subsets of the study cohort using the primary multivariable model. In the first subset, we included only admissions of six principal disease groups (see Additional file 1: Table S1). In the second subset, only admissions with no life-sustaining treatment limitations before transfer were included and, in the third subset, we excluded ICU-to-ICU transfers within the same hospital.

The regression results are reported as odds ratios (ORs) with 95% CI. We used STATA/SE 16 (StataCorp, College Station, TX, USA) for data analysis. A *p*-value < 0.05 was considered significant. This manuscript is conducted in accordance with the Equator network STROBE-statement [8].

## Results

**Fig. 1:**
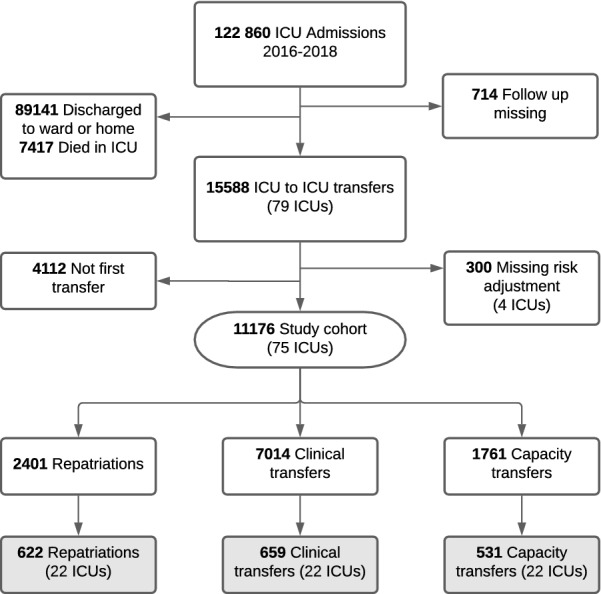
Patient flow diagram. The primary analysis included 11,176 transfers from 75 ICUs (bold boxes). A secondary analysis was based on 1812 transfers from 22 referring ICUs (grey boxes) where patients had a SOFA score recorded at discharge from the referring ICU

From 1st Jan, 2016 to 31st Dec, 2018 there were 112,860 adult admissions to Swedish ICUs, 15,588 (14.8%) of which ended with discharge by transfer to another ICU (see patient flow diagram Fig. 1). The majority were transferred for specialised care that was not available at the admitting hospital or ICU (clinical transfers, 62.8% of the study cohort). Capacity transfers were 15.8% of all transfers accounting for roughly 2.0% of ICU survivors.

Table 1 provides an overview of baseline patient data and characteristics of the referring ICUs. Clinical transfers differed from non-clinical transfers; they were slightly younger and less often male. Repatriation and capacity transfers were usually from tertiary care hospital ICUs where the median stay on the referring ICU was about 48 h before transfer. Most repatriations occurred during the day while almost one in five capacity transfers were at night. Capacity transfers had greater SOFA scores on discharge from the referring ICU, mainly due to cardiovascular and/or respiratory failure. Clinical transfers were more likely to have central nervous system injury while capacity transfers had acute lung injury and sepsis as primary diagnoses (see Additional file 1: Tables S2 and S3).

**Table 1:** Baseline patient data and characteristics of the referring ICU

	Repatriation *n* = 2401	Clinical transfers *n* = 7014	Capacity transfers *n* = 1761
Age, years mean (SD)	60.3 (17.9)	55.5 (18.9)	63.1 (16.4)
Male, *n* (%)	1536 (64.0)	4119 (58.7)	1097 (62.3)
Year of admission, *n* (% within year)			
2016	747 (19.9)	2443 (65.1)	565 (15.1)
2017	811 (22.2)	2282 (62.4)	563 (15.4)
2018	843 (22.4)	2289 (60.8)	633 (16.8)
Hospital category of referring ICU, *n* (% of category)			
Local hospital	368 (15.3)	2819 (40.2)	165 (9.4)
District general hospital	623 (26.0)	2905 (41.4)	475 (27.0)
Tertiary care hospital	1410 (58.7)	1290 (18.4)	1121 (63.7)
Time in hospital before ICU admission days, mean (SD)	2.7 (8.8)	1.6 (20.5)	3.0 (8.3)
Length of stay on referring ICU, hours; median (IQR)	47.6 (21.8–107.9)	11.8 (3.1–32.0)	48.2 (16.1–125.8)
Source of admission to referring ICU, *n* (% within category)			
Emergency room	771 (32.1)	4548 (64.8)	699 (39.7)
Theatre or PACU	425 (17.7)	457 (6.5)	236 (13.4)
Ward	441 (18.4)	1654 (23.6)	696 (39.5)
Other hospital or ICU	721 (30.0)	270 (3.9)	119 (6.8)
Other source	43 (1.8)	85 (1.2)	11 (0.6)
Surgical status on referring ICU, *n* (% within category)			
Elective surgery	193 (8.0)	164 (2.3)	107 (6.1)
Emergency surgery	440 (18.3)	475 (6.8)	223 (12.7)
Without surgery	1768 (73.6)	6375 (90.9)	1431 (81.3)
Illness severity (SAPS3) on admission to referring ICU, score, mean (SD)^a^			
SAPS3 score Box 1	7.9 (4.7)	7.0 (3.8)	8.3 (4.8)
SAPS3 score Box 2	27.7 (5.6)	27.7 (4.7)	28.5 (5.2)
SAPS3 score Box 3	12.4 (10.1)	10.8 (9.9)	16.9 (10.1)
SAPS3 total	56.9 (14.7)	53.0 (14.5)	63.5 (14.6)
SOFA-score at discharge, mean (SD)^b^	4.9 (3.5)	6.0 (4.1)	6.5 (3.6)
Time and day of discharge, *n* (% of category)			
Night-time discharge	112 (4.7)	1289 (18.4)	333 (18.9)
Weekend discharge	467 (19.5)	1777 (25.3)	394 (22.4)
Mortality			
30 days after ICU discharge, *n* (%)	390 (16.2)	1027 (14.6)	441 (25.0)
180 days after ICU discharge, *n* (%)	561 (23.4)	1468 (20.9)	584 (33.2)

^a^ See Methods for SAPS3 boxes

^b^The number of admissions with SOFA scores were for repatriation 622, clinical transfers 659 and capacity transfers 531

^c^For ICD-10 codes included in disease groups please see Additional file 1: Table S1

PACU: post-anaesthesia care unit; SAPS3: Simplified Acute Physiology Score version 3; SOFA: Sequential Organ Failure Assessment; ICH: intracranial haemorrhage; COPD: chronic obstructive pulmonary disease; ICU: intensive care unit

**Fig. 2:**
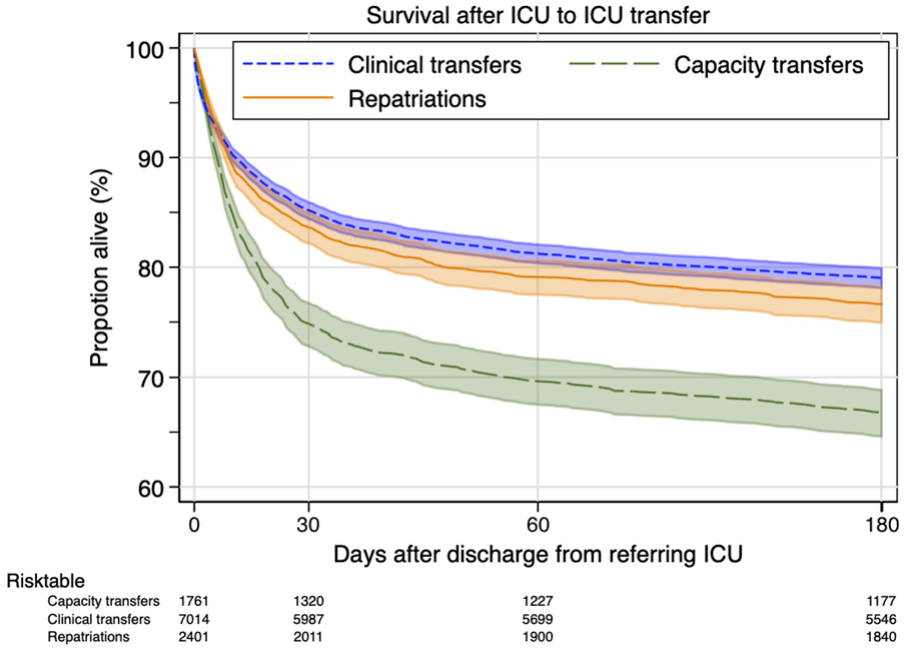
Kaplan–Meier diagram showing survival after ICU-to-ICU transfer. Shaded area showing 95% CI. Log-rank test *p* < 0.001

Unadjusted mortality within 30 days after discharge from the referring ICU was greater among capacity transfers where 25.0% died within 1 month of discharge (Table 1). Mortality in the capacity transfer group remained significantly higher for at least 180 days after discharge from the referring ICU, as seen in the unadjusted Kaplan–Meier diagram (Fig. 2).

Table 2 displays the uni- and multivariable associations between category of transfer and the primary end-point. In the primary multivariable analysis, an increased risk of death within 30 days of discharge was seen if the transfer was due to any other reason than repatriation. Adjusted results were similar when 180-day survival was analysed, with an OR of 1.19 (95% CI 1.02–1.39) *p* = 0.029 (see Additional file 1: Table S4).

**Table 2:** Association between covariates and 30-day mortality

	Single explanatory variable *n* = 11,176			Multivariable, all variables below included in analysis. *n* = 11,176		
	Odds ratio	95% CI	*p*-value	Odds ratio	95% CI	*p*-value
Type of transfer						
Repatriation	Reference			Reference		
Clinical transfer	0.88	0.78–1.00	.058	1.17	1.02–1.36	.029
Capacity transfer	1.72	1.48–2.00	< .001	1.25	1.06–1.49	.009
Variables adjusted for						
Age (per year)	1.04	1.04–1.05	< .001	1.04	1.04–1.05	< .001
Gender						
Female	Reference			Reference		
Male	1.12	1.01–1.24	.035	0.97	0.87–1.09	.627
SAPS3 score						
Box 1 (per point)^a^	1.09	1.08–1.10	< .001	1.06	1.05–1.07	< .001
Box 2 (per point)	1.05	1.04–1.06	< .001	1.03	1.02–1.04	< .001
Box 3 (per point)	1.07	1.06–1.07	< .001	1.06	1.06–1.07	< .001
Time and day of discharge						
Daytime	Reference			Reference		
Night-time	1.21	1.06–1.38	.004	1.20	1.04–1.40	.014
Weekday	Reference			Reference		
Weekend	0.92	0.82–1.04	.197	0.98	0.86–1.11	.730
ICU length of stay (per hour)	1.00	1.00–1.00	.002	1.00	1.00–1.00	.051
Principal disease group^b^						
Central nervous system injury	Reference			Reference		
COPD^c^	1.42	0.85–2.38	.178	1.09	0.65–1.85	.727
Cardiac arrest	2.77	2.18–3.51	< .001	1.56	1.20–2.01	.001
Acute lung injury	1.43	1.21–1.69	< .001	1.15	0.98–1.35	.072
Sepsis	1.27	1.04–1.55	.017	0.90	0.74–1.09	.292
Multi-trauma	0.34	0.24–0.47	< .001	0.72	0.51–1.02	.066
Other diagnoses	0.71	0.62–0.82	< .001	0.70	0.60–0.83	< .001

^a^Age deducted from score (see Methods)

^b^See Additional file 1: Table S1 for details of groups

^c^Chronic obstructive pulmonary disease

The results remained when adjusting for persistent organ failure at ICU discharge using SOFA scores. The 30-day mortality rate after ICU discharge was roughly 50% greater among those transferred for clinical or capacity reasons compared to repatriation (see Table 3). The increased risk associated with capacity transfer was slightly less pronounced when 180-day survival was analysed among admissions with SOFA score (adjusted OR 1.53, 95% CI 1.13–2.06, *p* = 0.006; see Additional file 1: Table S5).

**Table 3:** Association between covariates and 30-day mortality

	Single explanatory variable *n* = 1812			Multivariable, all variables below *n* = 1812			Multivariable, including SOFA-score *n* = 1812		
	Odds ratio	95% CI	*p*-value	Odds ratio	95% CI	*p*-value	Odds ratio	95% CI	*p*-value
Type of transfer									
Repatriation	Reference			Reference			Reference		
Clinical transfer	1.37	1.02–1.84	.035	1.77	1.27–2.48	.001	1.52	1.01–2.12	.014
Capacity transfer	2.21	1.66–2.97	< .001	1.71	1.22–2.39	.002	1.62	1.16–2.26	.005
Variables adjusted for									
Age (per year)	1.04	1.04–1.05	< .001	1.04	1.03–1.05	< .001	1.04	1.03–1.05	< .001
Gender									
Female	Reference			Reference			Reference		
Male	1.31	1.03–1.67	.027	1.19	0.92–1.55	.190	1.19	0.91–1.56	.200
SAPS3 score									
Box 1 (per point)^a^	1.06	1.04–1.09	< .001	1.05	1.02–1.07	.001	1.04	1.01–1.07	.006
Box 2 (per point)	1.04	1.02–1.06	< .001	1.04	1.02–1.07	.002	1.03	1.00–1.06	.032
Box 3 (per point)	1.05	1.04–1.06	< .001	1.04	1.03–1.06	< .001	1.03	1.02–1.04	< .001
Time and day of discharge									
Daytime	Reference			Reference			Reference		
Night-time	1.40	1.00–1.96	.050	1.19	0.82–1.73	.361	1.13	0.78–1.65	.515
Weekday	Reference			Reference			Reference		
Weekend	0.98	0.74–1.31	.913	0.93	0.68–1.26	.632	0.94	0.68–1.28	.682
ICU length of stay (per hour)	1.00	1.00–1.00	.519	1.00	1.00–1.00	.992	1.00	1.00–1.00	.469
Principal disease group^b^									
Central nervous system injury	Reference			Reference			Reference		
COPD^c^	0.76	0.22–2.70	.178	0.68	0.18–2.52	.566	0.65	0.17–2.47	.527
Cardiac arrest	2.41	1.44–4.04	< .001	1.08	0.57–2.03	.817	0.97	0.51–1.83	.923
Acute lung injury	1.27	0.89–1.82	< .001	0.79	0.52–1.21	.281	0.71	0.46–1.09	.116
Sepsis	1.80	1.16–1.55	.017	0.92	0.56–1.50	.735	0.71	0.43–1.17	.174
Multi-trauma	0.41	0.24–2.77	< .001	0.59	0.28–1.24	.167	0.59	0.28–1.23	.160
Other diagnoses	0.79	0.21–1.12	.186	0.71	0.48–1.05	.087	0.67	0.45–1.00	.050
SOFA-score on discharge									
Total score (per point)	1.20	1.17–1.24	< .001		Not included		1.15	1.11–1.19	< .001

1812 patients with complete SOFA-score on day of transfer

^a^ Age deducted from score (see Methods)

^b^See Additional file 1: Table S1 for details of groups

^c^Chronic obstructive pulmonary disease

We also applied our primary multivariable model in three subsets of the study cohort: patients belonging to any of six principal disease groups, patients with no life-sustaining treatment limitations before transfer and patients who were transferred to an ICU located in another hospital. The results were comparable to the analyses of the main study cohort as shown in Additional file 1: Tables S6, S7 and S8.

## Discussion

There were two principal findings in this study. First, non-clinical transfers due to resource constraints in the transferring ICU (capacity transfers) were associated with increased 30-day mortality compared to other non-clinical transfers (repatriations). Second, the proportion of ICU-to-ICU transfers in Sweden was greater than generally reported from comparable healthcare systems abroad. Before considering the implications of these findings, we need to discuss some methodological issues.

Understanding the impact of ICU-to-ICU transfer on patient outcome is complex and must consider a couple of important aspects. First, ICU-to-ICU transfers can be analysed from two different perspectives: the perspective of the referring ICU or the perspective of the receiving ICU. Second, whether a patient is transferred due to a clinical need or non-clinical reason must be known. Clinical transfers occur when there is a need for specialised care not available at the referring hospital. These transfers are usually associated with specific and sometimes lifesaving treatments, such as acute neurosurgical or cardiac interventions, which makes it difficult to find suitable non-transferred control patients. Hence, the first step must be to examine clinical and non-clinical transfers separately. Furthermore, non-clinical transfers need to be grouped into repatriations or capacity transfers, which usually have different urgencies. While the above categorisation facilitates an overall analysis, relevant control patients must still be identified for comparison of outcomes of non-clinical transfers. Studies from the referral’s perspective usually compare outcome of transferred patients with those staying in the ICU [3, 9], while studies from the receiver’s point-of-view usually compare outcomes of received referrals with those of admissions from the hospital’s own ward or emergency department [2, 10, 11]. Identifying appropriate control patients is challenging since non-clinical transfers are rarely undertaken when caregivers believe that discharge is imminent, either due to presumed recovery or death. Thus, comparing outcomes of patients transferred for non-clinical reasons with non-transferred patients may lead to biased results depending on the choice of control population.

In the present study, we examined transfers from the perspective of the referring hospital, and tried to circumvent the problem of identifying a proper control population by comparing outcomes within the cohort of transferred patients. This study design was based on the notion that, within the group of transferred patients, repatriations should be associated with the smallest adjusted risk of death due to ICU-to-ICU transfer. Repatriation was therefore used as our reference. Ideally, capacity transfers should also be associated with no or minimal risk increase compared to repatriations, while it is reasonable to believe that clinical transfers are associated with a higher risk.

Somewhat surprisingly, we found that 30-day mortality after capacity transfer was greater than after clinical transfer and repatriation in the unadjusted analyses. The difference in outcome between clinical and capacity transfers disappeared in the adjusted analyses but remained for repatriation. The adjusted risk relationships also remained after limiting analysis to admissions with information on organ failure (SOFA-score) at discharge in the multivariable analyses. While SOFA-score at discharge from the referring ICU was independently associated with poor outcome, it only partly explained the increased risk associated with clinical and capacity transfers compared to repatriation. Hence, other explanations not apparent from the results of the present study, must be considered.

It has been well established that transfer of critically ill patients is associated with more adverse outcomes partly due to transportation and poor communication of vital information [12–15]. In up to 50% of adverse events en route, pretransport recommendations provided by the referring intensivist were ignored [16]. Several studies have addressed the need for structured informative handover of the critically ill patient [17, 18]. However, further studies are needed to see if inadequacy of communication has less impact on the outcome of repatriation cases compared to clinical and capacity transfers. A more obvious explanation is that patients transferred for clinical and capacity reasons are more likely to endure an extra transfer compared to repatriations. A complete understanding of the care trajectory of patients undergoing ICU-to-ICU transfer is of great importance if we are to improve the chance of survival, particularly since such transports are likely to increase in the future [19].

The second notable result was that almost 15% of discharges from ICU were transfers to another ICU. Moreover, roughly 2% of all discharges were referred to another ICU due to lack of resources (capacity transfers). These numbers appear high compared to the literature [3, 20, 21], although most studies typically report transfers as a proportion of admissions to, rather than discharges from ICU. The overall high numbers of transfers may partly be explained by centralisation of specialised care to the few highly populated centres in our otherwise sparsely populated country. However, we believe that the large numbers of ICU-to-ICU capacity transfers also reflects the low overall number of available ICU beds in Sweden; one of the lowest in Europe [22, 23]. The high rate of capacity transfers suggest that ICUs regularly deliver care close to their surge capacity and prefer to transfer already admitted patients instead of refusing new patients with urgent needs. While refusal rates would enrich our analysis, such data were not available in the registry.

### Strengths and limitations

An important strength in this large nationwide study is that transfers were identified and categorised on discharge by ICU staff according to specific SIR guidelines. The validity of this categorisation is supported by the fact that the direction of clinical transfers was mainly from local and district general hospitals to tertiary care hospitals while non-clinical transfers were in the opposite direction. Clinical transfers were, as expected, younger with shorter length of stay in hospital and in ICU before transfer, also supporting that clinical and non-clinical transfers were grouped accurately. Within the non-clinical transfer group, the proper separation of repatriations and capacity transfers is supported by greater SOFA scores and more frequent night-time discharges among capacity transfers compared to repatriations. Another important strength was that the results remained largely the same when we limited our analysis to three specific subsets of our study cohort. Capacity transfers were associated with increased 30-day mortality whether analysed in six defined diagnostic groups, in admissions with no life-sustaining treatment limitations or after exclusion of transfers within the same hospital.

A significant limitation is the lack of data collected during transport and after arrival to the receiving ICU. Although we have information on 180-day survival, we need to know more if we are to understand how transfer influences outcome. An important next step must be to analyse and compare complete care trajectories of transferred and non-transferred patients. Linking ICU care episodes are needed to identify factors that are associated with survival, i.e. duration and mode of transport, organ support during transport, interventions or treatment limitations in the receiving ICU.

## Conclusion

This study identified an increased mortality rate associated with ICU-to-ICU transfers during periods of demand–supply mismatch. While prior studies have suggested that the only disadvantage of capacity transfers is longer intensive care periods [2, 3], this study shows that such transfers are also associated with greater mortality compared to repatriations. Avoiding the need for capacity transfers by increasing the number of ICU beds and staff is an obvious remedy. However, transfers due to demand–supply mismatch will continue to be necessary since it is impossible to meet all peaks in intensive care requirements. Future studies are needed to examine whether it is possible to minimise risk by careful patient selection, proper organisation of handover and transport and subsequent care in the receiving ICU.

## Supplementary Information

**Additional file 1.** Additional Tables.

## Data Availability

All data underlying this article were, after ethical approval, extracted and used unmodified from the Swedish Intensive Care Registry (SIR). The database cannot be shared publicly due to regulations under the Swedish law. Requests regarding the data may be made to the corresponding author.
