# Supplementary material for: Intensive care unit to unit capacity transfers are associated with increased mortality: an observational cohort study on patient transfers in the Swedish Intensive Care Register
Source: Ann Intensive Care. 2022 Apr 4;12:31. doi: 10.1186/s13613-022-01003-x (PMC8980179; doi:10.1186/s13613-022-01003-x)
Supplement: Supplementary file 1 — Additional file 1. Additional Tables. [file 13613_2022_1003_MOESM1_ESM.docx]

# **Supplementary Tables, Additional file 1.**

# Intensive care unit to unit capacity transfers are associated with increased mortality:

# an observational cohort study on patient transfers in the Swedish Intensive Care Register

Fredric Parenmark, Sten M. Walther

**Supplementary Table 1. ICD 10 groupings.**

| **Disease group** | **ICD-10 codes in group ^a^** |
| --- | --- |
| Central nervous system injury | S06.x, I60.x, I61.x, I63.x |
| COPD | J44.x |
| Cardiac arrest | I46.x |
| Acute lung injury | J12.x, J15.x, J69.x, J80.9, J81.9, J95.x |
| Sepsis | A41.x, R65.1, R57.2 |
| Multi-trauma | T07.9 |
| Other diagnoses | All other codes of the limited code set ^a^ |

^a^ The Swedish intensive care register used a reduced number of ICD-10 codes.

**Supplementary Table 2.**

**Principal disease group, n (%)**

| **Principal disease group** | **Repatriation n=2401** | **Clinical transfers n=7014** | **Capacity transfers**  **n=1761** |
| --- | --- | --- | --- |
| Central nervous system injury | 467 (19.5%) | 1,369 (19.5%) | 130 (7.4%) |
| COPD | 39 (1.6%) | 21 (0.3%) | 26 (1.5%) |
| Cardiac arrest | 113 (4.7%) | 173 (2.5%) | 97 (5.5%) |
| Acute lung injury | 427 (17.8%) | 620 (8.8%) | 576 (32.7%) |
| Sepsis | 169 (7.0%) | 503 (7.2%) | 225 (12.8%) |
| Multi-trauma | 173 (7.2%) | 379 (5.4%) | 47 (2.7%) |
| Other diagnoses | 1,013 (42.2%) | 3,949 (56.3%) | 660 (37.5%) |

**Supplementary Table 3.**

**SOFA score at discharge from ICU, mean (SD).**

| **Organ system** | **Repatriation**  **n=622** | **Clinical transfers n=659** | **Capacity transfers**  **n=531** |
| --- | --- | --- | --- |
| Respiratory | 1.9 (1.3) | 1.9 (1.4) | 2.1 (1.2) |
| Coagulation | 0.3 (0.6) | 0.5 (0.9) | 0.4 (0.8) |
| Hepatic | 0.3 (0.7) | 0.3 (0.7) | 0.3 (0.6) |
| Renal | 0.5 (1.1) | 0.7 (1.3) | 0.7 (1.2) |
| Cerebral | 1.0 (1.3) | 1.1 (1.4) | 1.2 (1.4) |
| Cardiovascular | 1.1 (1.5) | 0.7 (1.3) | 0.7 (1.2) |
| Total SOFA score | 4.9 (3.5) | 6.0 (4.1) | 6.5 (3.6) |

**Supplementary Table 4.**

**Association between covariates and 180-day mortality**

|  | **Single explanatory variable,**  **n=11,176** | | | **Multivariable, all variables below included,**  **n=11,176** | | |
| --- | --- | --- | --- | --- | --- | --- |
|  | Odds ratio | 95% CI | p-value | Odds ratio | 95% CI | p-value |
| **Type of transfer** |  |  |  |  |  |  |
| Repatriations | Reference |  |  | Reference |  |  |
| Clinical transfers | 0.87 | 0.78-0.97 | .012 | 1.20 | 1.06-1.37 | .004 |
| Capacity transfers | 1.63 | 1.42-1.87 | <.001 | 1.19 | 1.02-1.39 | .029 |
|  |  |  |  |  |  |  |
| **Variables adjusted for** |  |  |  |  |  |  |
| **Age (per year)** | 1.05 | 1.04-1.05 | <.001 | 1.05 | 1.04-1.05 | <.001 |
| **Gender** |  |  |  |  |  |  |
| Female | Reference |  |  | Reference |  |  |
| Male | 1.12 | 1.03-1.23 | .011 | 1.00 | 0.90-1.10 | .980 |
| **SAPS3 score** |  |  |  |  |  |  |
| Box 1 (per point) ^a^ | 1.12 | 1.11-1.13 | <.001 | 1.09 | 1.08-1.10 | <.001 |
| Box 2 (per point) | 1.05 | 1.04-1.06 | <.001 | 1.03 | 1.02-1.04 | <.001 |
| Box 3 (per point) | 1.06 | 1.05-1.06 | <.001 | 1.05 | 1.05-1.06 | <.001 |
| **Time and day of discharge** |  |  |  |  |  |  |
| Daytime | Reference |  |  | Reference |  |  |
| Night-time | 1.13 | 1.00-1.27 | .044 | 1.13 | 1.00-1.30 | .066 |
| Weekday | Reference |  |  | Reference |  |  |
| Weekend | 0.90 | 0.81-1.00 | .053 | 0.98 | 0.87-1.10 | .739 |
| ICU length of stay (per hour) | 1.00 | 1.00-1.00 | <.001 | 1.00 | 1.00-1.00 | .549 |
| **Principal disease group ^b^** |  |  |  |  |  |  |
| Central nervous system injury | Reference |  |  | Reference |  |  |
| COPD ^c^ | 1.50 | 0.94-2.38 | .087 | 0.80 | 0.49-1.31 | .379 |
| Cardiac arrest | 2.18 | 1.74-2.74 | <.001 | 0.96 | 0.73-1.26 | .769 |
| Acute Lung Injury | 1.55 | 1.34-1.79 | <.001 | 0.88 | 0.74-1.04 | .140 |
| Sepsis | 1.32 | 1.10-1.57 | .002 | 0.65 | 0.53-0.79 | <.001 |
| Multi-trauma | 0.34 | 0.25-0.45 | <.001 | 0.52 | 0.38-0.72 | <.001 |
| Other diagnoses | 0.73 | 0.65-0.84 | <.001 | 0.73 | 0.63-0.84 | <.001 |
| **SOFA-score on discharge ^d^** |  |  |  |  |  |  |
| Total score (per point) | 1.17 | 1.14-1.20 | <.001 |  | Not included |  |

^a^ Age deducted from score (see methods), ^b^ See supplementary material table 1 for details of groups, ^c^ Chronic obstructive pulmonary disease,

^d^ n=1,812 patients with SOFA-score at discharge from ICU

**Supplementary Table 5.**

**Association between covariates and 180-day mortality for patients with SOFA-score**

|  | **Multivariable, all variables below included,**  **n=1,812** | | |
| --- | --- | --- | --- |
|  | Odds ratio | 95% CI | p-value |
| **Type of transfer** |  |  |  |
| Repatriations | Reference |  |  |
| Clinical transfers | 1.32 | 0.99-1.78 | .063 |
| Capacity transfers | 1.53 | 1.13-2.06 | .006 |
|  |  |  |  |
| **Variables adjusted for** |  |  |  |
| **Age (per year)** | 1.04 | 1.03-1.05 | <.001 |
| **Gender** |  |  |  |
| Female | Reference |  |  |
| Male | 1.26 | 0.98-1.59 | .073 |
| **SAPS3 score** |  |  |  |
| Box 1 (per point) ^a^ | 1.06 | 1.03-1.09 | <.001 |
| Box 2 (per point) | 1.03 | 1.01-1.06 | .010 |
| Box 3 (per point) | 1.03 | 1.01-1.04 | <.001 |
| **Time and day of discharge** |  |  |  |
| Daytime | Reference |  |  |
| Night-time | 1.14 | 0.80-1.62 | .462 |
| Weekday | Reference |  |  |
| Weekend | 1.15 | 0.87-1.52 | .332 |
| ICU length of stay (per hour) | 1.00 | 1.00-1.00 | .451 |
| **Principal disease group ^b^** |  |  |  |
| Central nervous system injury | Reference |  |  |
| COPD ^c^ | 0.89 | 0.29-2.72 | .841 |
| Cardiac arrest | 0.92 | 0.51-1.69 | .799 |
| Acute lung injury | 0.81 | 0.55-1.20 | .300 |
| Sepsis | 0.76 | 0.48-1.21 | .247 |
| Multi-trauma | 0.72 | 0.38-1.35 | .305 |
| Other diagnoses | 0.78 | 0.54-1.11 | .166 |
| **SOFA-score on discharge** |  |  |  |
| Total score (per point) | 1.12 | 1.08-1.16 | <.001 |

^a^ Age deducted from score (see methods), ^b^ See supplementary material table 1 for details of groups,

^c^ Chronic obstructive pulmonary disease

**Supplementary Table 6.**

**Association between covariates and 30-day mortality for patients from six principal disease groups only.**

|  | **Multivariable, all variables below included.**  **n=5,554** | | |
| --- | --- | --- | --- |
|  | Odds ratio | 95% CI | p-value |
| **Type of transfer** |  |  |  |
| Repatriation | Reference |  |  |
| Clinical transfer | 1.15 | 0.95-1.38 | .153 |
| Capacity transfer | 1.35 | 1.10-1.67 | .005 |
|  |  |  |  |
| **Variables adjusted for** |  |  |  |
| **Age (per year)** | 1.04 | 1.03-1.05 | <.001 |
| **Gender** |  |  |  |
| Female | Reference |  |  |
| Male | 0.96 | 0.82-1.05 | .554 |
| **SAPS3 score** |  |  |  |
| Box 1 (per point) ^a^ | 1.06 | 1.05-1.08 | <.001 |
| Box 2 (per point) | 1.02 | 1.00-1.03 | .018 |
| Box 3 (per point) | 1.06 | 1.05-1.07 | <.001 |
| **Time and day of discharge** |  |  |  |
| Daytime | Reference |  |  |
| Night-time | 1.13 | 0.93-1.38 | .227 |
| Weekday | Reference |  |  |
| Weekend | 0.98 | 0.82-1.16 | .807 |
| ICU length of stay (per hour) | 1.00 | 1.00-1.00 | .049 |
| **Principal disease group ^b^** |  |  |  |
| Central nervous system injury | Reference |  |  |
| COPD ^c^ | 0.74 | 1.19-3.28 | .276 |
| Cardiac arrest | 1.07 | 0.80-1.44 | .659 |
| Acute lung injury | 0.79 | 0.64-0.97 | .023 |
| Sepsis | 0.63 | 0.50-0.79 | .001 |
| Multi-trauma | 0.48 | 0.34-0.70 | .001 |
|  |  |  |  |

^a^ Age deducted from score (see methods),  ^b^ See supplementary material table 1 for details of groups, ^c^ Chronic obstructive pulmonary disease.

**Supplementary table 7.**

**Association between covariates and 30-day mortality for patients with no limitations of care**

|  | **Multivariable, all variables below included**  **n=7,423** | | |
| --- | --- | --- | --- |
|  | Odds ratio | 95% CI | p-value |
| **Type of transfer** |  |  |  |
| Repatriations | Reference |  |  |
| Clinical transfers | 1.32 | 1.08-1.60 | .006 |
| Capacity transfers | 1.32 | 1.05-1.66 | .017 |
|  |  |  |  |
| **Variables adjusted for** |  |  |  |
| **Age (per year)** | 1.04 | 1.04-1.05 | <.001 |
| **Gender** |  |  |  |
| Female | Reference |  |  |
| Male | 0.91 | 0.79-1.05 | .204 |
| **SAPS3 score** |  |  |  |
| Box 1 (per point) ^a^ | 1.06 | 1.05-1.08 | <.001 |
| Box 2 (per point) | 1.02 | 1.01-1.04 | .003 |
| Box 3 (per point) | 1.06 | 1.05-1.07 | <.001 |
| **Time and day of discharge** |  |  |  |
| Daytime | Reference |  |  |
| Night-time | 1.22 | 1.01-1.48 | .040 |
| Weekday | Reference |  |  |
| Weekend | 1.03 | 0.87-1.22 | .722 |
| ICU length of stay (per hour) | 1.00 | 1.00-1.00 | .092 |
| **Principal disease group ^b^** |  |  |  |
| Central nervous system injury | ref |  |  |
| COPD ^c^ | 0.56 | 0.25-1.28 | .170 |
| Cardiac arrest | 0.84 | 0.57-1.22 | .351 |
| Acute lung injury | 0.81 | 0.63-1.04 | .010 |
| Sepsis | 0.63 | 0.77-1.32 | .960 |
| Multi-trauma | 0.50 | 0.47-0.84 | .001 |
| Other diagnoses | 0.67 | 0.54-0.83 | <.001 |

^a^ Age deducted from score (see Methods), ^b^ See supplementary material table 1 for details of groups, ^c^ Chronic obstructive pulmonary disease

**Supplementary table 8.**

**Association between covariates and 30-day mortality for patients transferred outside their referring hospital.**

|  | Multivariable, all variables below included  n=7,178 | | |
| --- | --- | --- | --- |
|  | Odds ratio | 95% CI | p-value |
| **Type of transfer** |  |  |  |
| Repatriations | Reference |  |  |
| Clinical transfers | 1.28 | 1.04-1.58 | .021 |
| Capacity transfers | 1.53 | 1.17-1.99 | .002 |
|  |  |  |  |
| **Variables adjusted for** |  |  |  |
| **Age (per year)** | 1.04 | 1.04-1.05 | <.001 |
| **Gender** |  |  |  |
| Female | Reference |  |  |
| Male | 0.86 | 0.75-1.00 | .045 |
| **SAPS3 score** |  |  |  |
| Box 1 (per point) ^a^ | 1.06 | 1.04-1.07 | <.001 |
| Box 2 (per point) | 1.03 | 1.01-1.04 | .002 |
| Box 3 (per point) | 1.07 | 1.06-1.07 | <.001 |
| **Time and day of discharge** |  |  |  |
| Daytime | Reference |  |  |
| Night-time | 1.26 | 1.05-1.51 | .015 |
| Weekday | Reference |  |  |
| Weekend | 1.09 | 0.92-1.28 | .317 |
| ICU length of stay (per hour) | 1.00 | 1.00-1.00 | .203 |
| **Principal disease group ^b^** |  |  |  |
| Central nervous system injury | ref |  |  |
| COPD ^c^ | 1.36 | 0.72-2.58 | .346 |
| Cardiac arrest | 1.14 | 0.79-1.64 | .498 |
| Acute lung injury | 1.17 | 0.94-1.45 | .164 |
| Sepsis | 0.90 | 0.70-1.15 | .396 |
| Multi-trauma | 0.56 | 0.35-0.91 | .019 |
| Other diagnoses | 1.46 | 1.19-1.78 | <.001 |

^a^ Age deducted from score (see Methods), ^b^ See supplementary material table 1 for details of groups, ^c^ Chronic obstructive pulmonary disease
